# Dieulafoy's Disease Causing Appendiceal Hemorrhage: A Case Report

**DOI:** 10.7759/cureus.89940

**Published:** 2025-08-12

**Authors:** Ruitao Liu, Yang Yan, Guibing Chen

**Affiliations:** 1 Surgery, Chengdu Medical College, Chengdu, CHN; 2 Gastrointestinal Surgery, The First Affiliated Hospital of Chengdu Medical College, Chengdu, CHN

**Keywords:** appendectomy, appendiceal bleeding, dieulafoy's disease, lower gastrointestinal bleeding, surgery

## Abstract

Dieulafoy’s disease is an uncommon but potentially life-threatening vascular anomaly characterized by a dilated, aberrant submucosal artery that causes mucosal erosion and significant gastrointestinal bleeding. While most frequently located in the proximal stomach, Dieulafoy’s lesions can occur anywhere in the GI tract. Involvement of the appendix is exceedingly rare and may pose a diagnostic challenge due to the inconspicuous nature of the lesion and its inaccessibility via routine endoscopy. We present a rare case of appendiceal Dieulafoy’s disease manifesting as isolated lower gastrointestinal bleeding.

## Introduction

Lower gastrointestinal bleeding (LGIB) is a common clinical challenge with a broad differential diagnosis, ranging from benign anorectal disorders to vascular malformations and neoplasms. While hemorrhoids, diverticulosis, and colorectal neoplasia represent the majority of causes, bleeding originating from the appendix is exceedingly rare, accounting for less than 0.5‰ of all LGIB cases [1,2]. Among the vascular etiologies, Dieulafoy’s lesion, defined as a submucosal artery that maintains an abnormally large diameter and penetrates the mucosal surface, is an especially uncommon source of appendiceal hemorrhage, with only a handful of cases reported to date [3-7]. Dieulafoy’s lesion was first described in the stomach, but it has subsequently been identified throughout the gastrointestinal tract, including the esophagus, duodenum, jejunum, colon, and appendix [8,9]. The pathogenesis involves mucosal erosion overlying a persistent, pulsatile artery, leading to massive or recurrent bleeding without apparently surrounding inflammation [8]. Due to its rarity, concealment, and diagnostic complexity, appendiceal Dieulafoy’s lesion constitutes a clinical emergency. In this report, we present a rare case of appendiceal Dieulafoy’s lesion in a young adult male. By discussing this case, we further summarize the diagnosis and management of appendiceal hemorrhage.

## Case presentation

**Figure 1:**
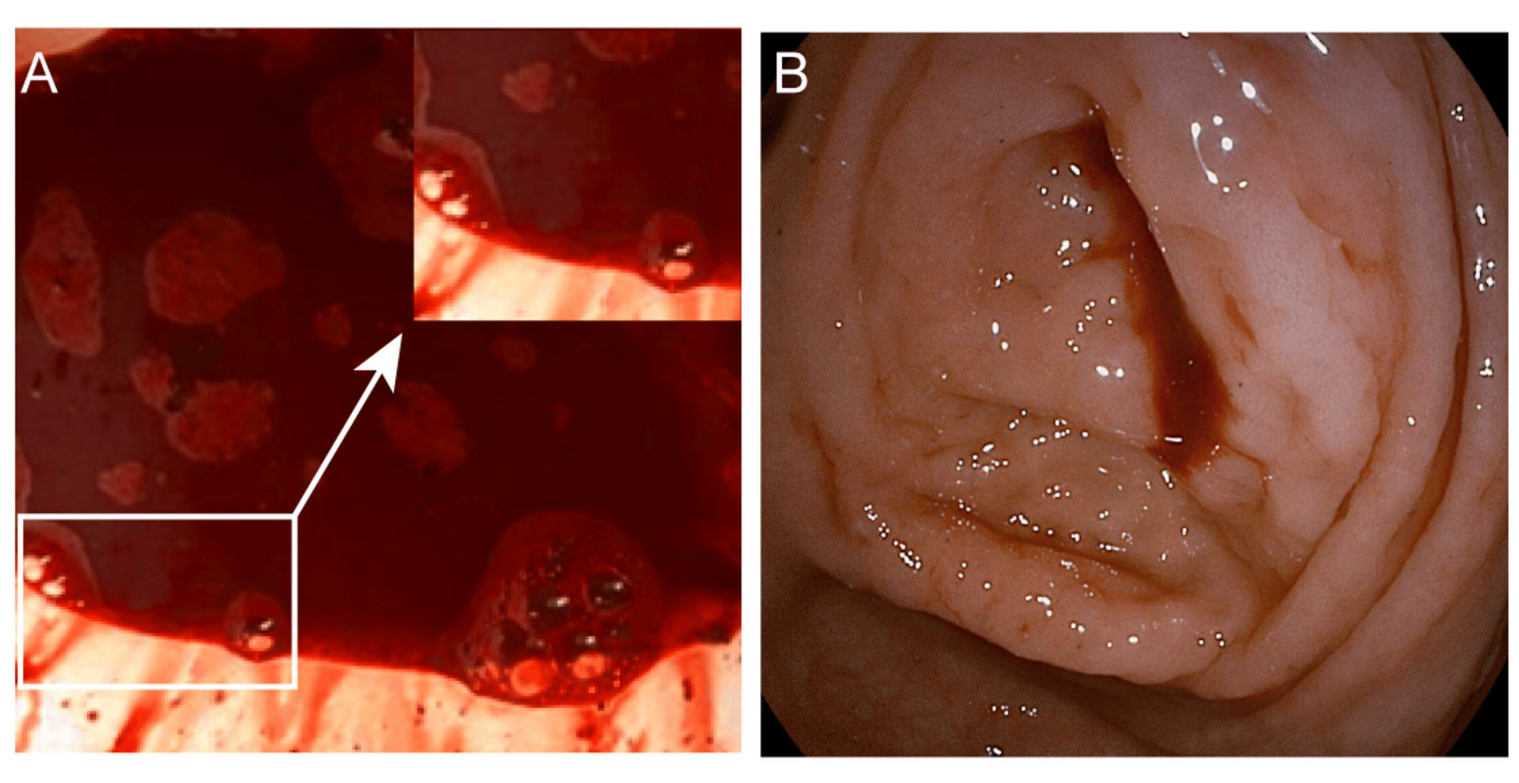
Clinical manifestation of the patient with appendiceal hemorrhage. A. The patient's bloody stools were dark red; B. Colonoscopy showed fresh blood extravasation from the appendiceal orifice.

A 26-year-old man presented to the emergency department with a four-day history of hematochezia. He reported passing dark red blood per rectum two to three times daily, without associated symptoms such as dizziness, palpitations, or fatigue (Figure 1A). His medical history was notable for internal hemorrhoids. On initial evaluation, a rectal examination revealed bleeding internal hemorrhoids. Treatment was initiated with lukewarm sitz baths and Ma Yinglong suppositories; however, symptoms persisted. The patient was subsequently transferred to the Gastroenterology Department for further workup. On admission, vital signs were within normal limits: temperature 36.5 °C, pulse 90 bpm, respiratory rate 18 breaths/min, and blood pressure 119/78 mmHg. The patient was alert and oriented. Cardiopulmonary examination was unremarkable. Abdominal examination revealed a soft abdomen with mild periumbilical tenderness and no signs of guarding. Digital rectal examination detected blood on the examining finger but no palpable masses. Anoscopy performed in the knee-chest position showed longitudinal mucosal bulges at 3, 5, 7, and 11 o’clock within the internal hemorrhoidal zone, with mild congestion and no evidence of erosion or active bleeding.

**Figure 2:**
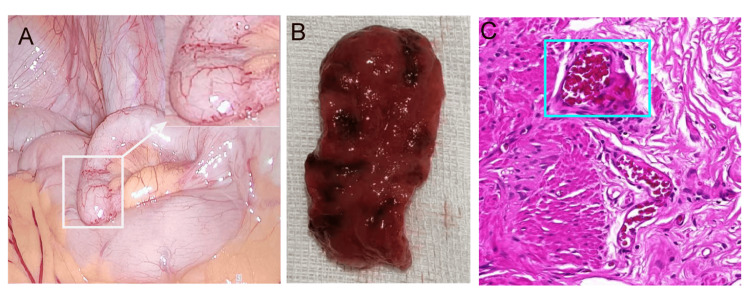
Surgical specimen of the patient with appendiceal hemorrhage. A. laparoscopy showed marked dilatation of blood vessels in the caudal portion of the appendiceal body; B. dissection of the appendiceal specimen showed punctate erosions of the mucosa accompanied by dark red hemorrhagic spots; C. postoperative pathology showed dilated and tortuous submucosal vascular diameters (X40, HE staining).

Laboratory investigations revealed: red blood cell count 3.59 × 10¹²/L (Normal:3.8-5.1×1012/L), hemoglobin 110 g/L (Normal:115-150 g/L), white blood cell count 4.00 × 10⁹/L (Normal:3.5-9.5×109/L), and neutrophils 2.40 × 10⁹/L (Normal:1.8-6.3×109/L), consistent with mild anemia. A preliminary diagnosis of lower gastrointestinal bleeding was made. The patient was started on fluid resuscitation, hemostatic therapy (somatostatin at a dose of 3 mg every 12 hours using an infusion pump), and acid suppression (esomeprazole sodium at a dose of 40 mg once daily). Bowel preparation was initiated in anticipation of colonoscopy. Colonoscopy demonstrated active spurting bleeding from the appendiceal orifice (Figure 1B), establishing the diagnosis of appendiceal hemorrhage. Despite conservative management including fluid replacement, hemostatic agents, and continuous somatostatin infusion, the patient continued to pass thin, bloody stools intermittently. The patient was subsequently transferred to the Department of Gastrointestinal Surgery for an emergency laparoscopic appendectomy under general anesthesia. Intraoperatively, the appendix appeared mildly edematous, measuring approximately 0.6 cm in diameter and 5 cm in length. Notable hyperplasia and dilation of the mesenteric vessels were observed at the distal end (Figure 2A). Examination of the resected specimen revealed intraluminal blood accumulation and multiple mucosal ulcers with associated hemorrhagic foci (Figure 2B). Histopathological analysis confirmed dilated, tortuous submucosal vessels of uniform diameter, consistent with an appendiceal Dieulafoy's lesion (Figure 2C). On postoperative day 1, the patient's general condition remained stable. Routine blood tests showed: RBC 4.17×10¹²/L (Normal:3.8-5.1×1012/L), hemoglobin 109 g/L (Normal:115-150 g/L), WBC 8.29×10⁹/L (Normal:3.5-9.5×109/L), and neutrophils 6.26×10⁹/L (Normal:1.8-6.3×109/L). By postoperative day 2, the patient resumed passing flatus and progressed from a low-residue to a regular diet. On postoperative days 4 and 6, he passed yellow, loose stools without visible blood. He was discharged on postoperative day 7. At a five-month telephone follow-up, the patient reported no recurrent bleeding, and laboratory parameters remained within normal limits (hemoglobin 121 g/L).

## Discussion

Overview of the disease

Dieulafoy’s disease, also known as Dieulafoy’s ulcer, is a rare cause of gastrointestinal bleeding resulting from rupture of a submucosal arterial malformation. The pathogenesis involves a large-caliber, aberrant artery that traverses the submucosa and maintains a constant diameter. Persistent pulsation of the artery compresses and atrophies the overlying mucosa, leading to the formation of superficial ulcers and eventual rupture. This condition predominantly affects the stomach [9]. To date, only five cases of appendiceal Dieulafoy’s disease [3-7] have been reported. When located in the appendix, it typically presents as dark-red hematochezia due to lower gastrointestinal bleeding. A retrospective study in China reported appendiceal hemorrhage as an exceedingly rare cause of lower gastrointestinal bleeding, accounting for only 0.14‰ of cases [1]. In addition to hematochezia, clinical manifestations may include nonspecific right lower quadrant pain or severe colic, often attributable to inflammatory stimulation of the appendix and intraluminal blood accumulation.

Diagnostic methods

Timely diagnosis of appendiceal hemorrhage remains a significant clinical challenge. Conventional imaging modalities, such as ultrasound and computed tomography (CT), often fail to detect the bleeding source, and colonoscopy requires prior bowel preparation, which can delay diagnosis. As a result, initial management typically involves conservative measures, including fluid resuscitation, administration of hemostatic agents, and, when necessary, blood transfusion. Colonoscopy is generally deferred until the patient's condition stabilizes. In the present case, definitive evidence of active appendiceal bleeding was obtained only after colonoscopy. The primary diagnostic difficulties associated with identifying appendiceal hemorrhage during colonoscopy include: (1) the narrow and easily overlooked appendiceal orifice, and (2) the potential absence of fresh blood or clots within the lumen, which may obscure active bleeding. Reports in the literature suggest that repeated irrigation of the appendiceal orifice and lumen can improve detection rates [7]. Additionally, because appendiceal bleeding is often intermittent, slow, or transient, multiple colonoscopic evaluations may be required to achieve a definitive diagnosis. Differential diagnosis should include other potential sources of lower gastrointestinal bleeding, particularly from the small intestine. Diagnostic modalities such as angiography [10] and capsule endoscopy can aid in distinguishing appendiceal hemorrhage from alternative etiologies. In this case, the initial diagnosis was internal hemorrhoidal bleeding, likely influenced by the patient's prior history of hemorrhoids.

A review of published case reports indicates that the etiology of appendiceal hemorrhage can be broadly categorized into three primary groups: (1) inflammation-related lesions-for instance, appendiceal bleeding may occur secondary to acute appendicitis [11]. One paper notes that luminal obstruction and subsequent bacterial invasion lead to mucosal ulceration and vascular injury, resulting in hemorrhage. Appendiceal bleeding has also been associated with conditions such as appendiceal diverticulitis and Crohn’s disease [12]. Collectively, these findings indicate that inflammatory processes are significant etiologic factors in appendiceal hemorrhage. (2) Tumor-related lesions: Some scholars described a case in which appendiceal bleeding was caused by mucinous papillary cystadenocarcinoma of the right ovary that had invaded the appendix, as identified during preoperative colonoscopy [13]. (3) Vascular-related lesions: Vascular abnormalities have also been implicated. A number of scholars reported cases involving vascular hyperplasia of the appendiceal mucosa [14,15], while various scholars [3-7] attributed appendiceal bleeding to a Dieulafoy’s lesion. In the present case, histopathological examination revealed punctate mucosal erosions with dark red hemorrhagic foci and dilated submucosal vessels of uniform diameter-findings consistent with an appendiceal Dieulafoy’s lesion. Evaluation of inflammatory markers and contrast-enhanced abdominal CT, in conjunction with the patient’s history and physical examination, demonstrated no evidence of inflammatory bowel disease or neoplasia.

Treatment and prognosis

Treatment options for gastrointestinal bleeding due to Dieulafoy’s lesions include endoscopic hemostasis [16,17], vascular embolization, and surgical intervention [18]. While endoscopic treatment is commonly employed for gastric lesions, its application in the appendix is limited due to the narrow lumen, which hinders access and increases the risk of iatrogenic appendicitis or recurrent bleeding [19]. Notably, in all five reported cases of appendiceal Dieulafoy lesions, hemostasis was successfully achieved through appendectomy [3-7]. Thus, appendectomy is considered the most definitive and effective treatment for appendiceal hemorrhage. The favorable clinical outcome in our patient reinforces this conclusion. Therefore, once appendiceal bleeding is diagnosed, prompt surgical intervention is strongly recommended.

## Conclusions

Appendiceal Dieulafoy’s disease is an exceptionally rare but important cause of lower gastrointestinal bleeding that should be considered when more common sources are excluded, particularly in patients with persistent hematochezia and inconclusive endoscopic findings. Due to the lesion's subtle presentation and the appendix's limited accessibility, diagnosis is often delayed. Colonoscopy, when performed during active bleeding, may aid in localization, but definitive diagnosis frequently relies on histopathological examination following surgical resection. As demonstrated in this case, laparoscopic appendectomy remains a safe and effective treatment modality, offering both diagnostic clarity and therapeutic resolution. Prompt surgical intervention upon identification of appendiceal hemorrhage is recommended to prevent ongoing blood loss and improve patient outcomes.
